# Data Augmentation and Transfer Learning for Data Quality Assessment in Respiratory Monitoring

**DOI:** 10.3389/fbioe.2022.806761

**Published:** 2022-02-14

**Authors:** Andrea Rozo, Jonathan Moeyersons, John Morales, Roberto Garcia van der Westen, Lien Lijnen, Christophe Smeets, Sjors Jantzen, Valerie Monpellier, David Ruttens, Chris Van Hoof, Sabine Van Huffel, Willemijn Groenendaal, Carolina Varon

**Affiliations:** ^1^ STADIUS Center for Dynamical Systems, Signal Processing and Data Analytics, Department of Electrical Engineering (ESAT), KU Leuven, Leuven, Belgium; ^2^ Microgravity Research Center, Service Chimie-Physique, Université Libre de Bruxelles, Brussels, Belgium; ^3^ Imec The Netherlands/Holst Centre, Eindhoven, Netherlands; ^4^ Department of Medicine and Life Sciences, Hasselt University, Diepenbeek, Belgium; ^5^ Future Health department, Pneumology department, Ziekenhuis Oost-Limburg, Genk, Belgium; ^6^ Nederlandse Obesitas Kliniek, Venlo, Netherlands; ^7^ Nederlandse Obesitas Kliniek, Huis ter Heide, Netherlands; ^8^ Imec OnePlanet, Wageningen, Netherlands; ^9^ Electronic Circuits and Systems (ECS), Department of Electrical Engineering (ESAT), KU Leuven, Leuven, Belgium; ^10^ Imec, Leuven, Belgium

**Keywords:** respiratory monitoring, signal quality, machine learning, transfer learning, data augmentation

## Abstract

Changes in respiratory rate have been found to be one of the early signs of health deterioration in patients. In remote environments where diagnostic tools and medical attention are scarce, such as deep space exploration, the monitoring of the respiratory signal becomes crucial to timely detect life-threatening conditions. Nowadays, this signal can be measured using wearable technology; however, the use of such technology is often hampered by the low quality of the recordings, which leads more often to wrong diagnosis and conclusions. Therefore, to apply these data in diagnosis analysis, it is important to determine which parts of the signal are of sufficient quality. In this context, this study aims to evaluate the performance of a signal quality assessment framework, where two machine learning algorithms (support vector machine–SVM, and convolutional neural network–CNN) were used. The models were pre-trained using data of patients suffering from chronic obstructive pulmonary disease. The generalization capability of the models was evaluated by testing them on data from a different patient population, presenting normal and pathological breathing. The new patients underwent bariatric surgery and performed a controlled breathing protocol, displaying six different breathing patterns. Data augmentation (DA) and transfer learning (TL) were used to increase the size of the training set and to optimize the models for the new dataset. The effect of the different breathing patterns on the performance of the classifiers was also studied. The SVM did not improve when using DA, however, when using TL, the performance improved significantly (*p* < 0.05) compared to DA. The opposite effect was observed for CNN, where the biggest improvement was obtained using DA, while TL did not show a significant change. The models presented a low performance for shallow, slow and fast breathing patterns. These results suggest that it is possible to classify respiratory signals obtained with wearable technologies using pre-trained machine learning models. This will allow focusing on the relevant data and avoid misleading conclusions because of the noise, when designing bio-monitoring systems.

## 1 Introduction

Spaceflights impose many challenges to the well-being of astronauts due to their particular conditions, such as altered gravity, radiation, isolation and confinement (Vernikos, 1996; Tascher et al., 2019). The effects of these conditions on the health of the crew include affections to bone and muscle structures as well as deregulation of metabolic, cardiovascular, respiratory and immunologic systems, among others (Tascher et al., 2019; Crucian et al., 2013, 2015). Combined with the limited access to medical attention, these alterations can be a high risk to the crew members during the missions.

In addition to systemic alterations, the crew members can suffer from injuries, wounds, traumatic events and surgical emergencies. Illnesses and infections may also be acquired even though, prior to the start of the spaceflight, the crew undergoes a medical screening and a quarantine period. These events can be associated with external environmental conditions, such as first-degree burns as a result of ultraviolet light exposure, or with the internal atmosphere of the spacecraft, such as latent viruses reactivation, higher number of free-floating particles, chemicals, allergens, and microorganisms (e.g., bacteria, fungi and molds) (Crucian et al., 2016; Barratt et al., 2020; Barrila et al., 2021).

While in past and current space missions the incidence of these issues has been low, this might not be the case for longer missions like Mars or asteroid exploration. In such missions, health care *in-situ* becomes even more critical as delays in communications with the Earth are more common, and emergency extraction of crew members that could fall ill are harder. Under these new circumstances, wounds and infections require special attention regarding their management and monitoring.

It has been observed that in spaceflight conditions, the healing process of wounds is altered (Cialdai et al., 2020). This, in conjunction with the dysfunction of the immune system might cause complications. During the healing process, the continuous monitoring of vital signs can help to identify changes in the state of the patient and, in this way, allows to take the appropriate measures when deterioration is discovered (Brown et al., 2014).

Wearable technology presents a suitable alternative to traditional monitoring for continuous measurement of vital signs, because it does not interfere with the mobility and comfort of the patient. In the last years, this technology has been evaluated in clinical environments on Earth (Fieselmann et al., 1993; Prgomet et al., 2016; Weenk et al., 2020; Subbe and Kinsella, 2018). For the case of space exploration, joint efforts between space agencies (National Aeronautics and Space Administration—NASA, Canadian Space Agency—CSA) and industry have resulted in the design of wearable sensors to monitor the vital signs of the crew members during missions, which have been tested in the International Space Station (ISS) and in settings on ground to validate their performance (Mundt et al., 2005; Bellisle et al., 2020; Falker et al., 2015; Villa-Colín et al., 2018).

Even though the continuous monitoring works appropriately and the versatility of the wearable devices allows a wide range of movements, it has been found that this added flexibility makes the recorded signals more prone to noise sources (Orphanidou et al., 2015). As a consequence, the presence of artefacts prevents the extraction of reliable information, increasing the probability of false alarms and inaccurate measures. Some of the artefacts can be easily removed through filtering, but others are beyond repair, such as motion artefacts due to displacement of electrodes, for example, as observed in Villa-Colín et al. (2018).

In order to overcome the difficulties related with the removal of artefacts, some works have implemented signal quality indication approaches to identify the parts of the data that are useful for analysis (Orphanidou et al., 2015; Johnson et al., 2015; Castro et al., 2016; Castro et al., 2018; Charlton et al., 2021; Moeyersons et al., 2021). In general, the quality indication for electrocardiogram and photoplethysmogram signals, for the estimation of heart rate, has been investigated further than the quality indication of other signals, such as respiration.

The respiratory signal, however, is of great interest in health monitoring, given that it is one of the key markers that indicates deterioration in patients. Increasing respiratory rate (i.e., going above 20 breaths per minute) has been found to be an early predictor in different life-threatening conditions, such as cardiac arrest, respiratory adverse events and sepsis (Cretikos et al., 2008; Lee, 2016; Hotchkiss et al., 2016). Nevertheless, when the respiratory rate is not overlooked, its monitoring is often performed manually and at specific times during the day. The use of wearable devices for this task is not yet widely spread, because of the lack of evidence of accurate and reliable results. Given the useful characteristics of this signal for determining health adverse conditions (Nicolò et al., 2020) and the importance of its continuous monitoring in the early detection of these situations, this study is focused in the quality assessment of respiratory signals obtained from wearable sensors.

Previously, a quality index for respiratory signals was developed by Charlton et al. (2021), which then was compared to a machine learning framework for quality assessment by Moeyersons et al. (2021). Rozo et al. (2021) presented the results of applying transfer learning to the previous framework. In this context, this study extends the latter work including a data augmentation approach to improve the performance of the machine learning framework when applied to new data.

The presented framework consists of two machine learning models, which are used to classify segments of thoracic bio-impedance (BioZ) signals into clean or noisy (containing artefacts). The first classification model is a support vector machine (SVM), and the second one is a convolutional neural network (CNN). These models were designed for a dataset of patients suffering from chronic obstructive pulmonary disease (COPD).

Currently, there is a lack of available data from space exploration missions, in which health adverse situations have occurred, thanks to the extensive training and health screenings of the astronauts previous to the flight, combined with a relatively short time in the mission. Considering this, and the fact that the framework presented in this study could be used for the monitoring of astronauts’ health during longer missions where pathological breathing could be observed, a “worst case scenario” is presented. To this end, multiple respiratory patterns are included by testing the models on patients’ data. As a consequence, the algorithms are tested not only on “normal” breathing patterns but they are also adequate for diseased conditions.

Considering this, the goal of this study is twofold. First, the models are pre-trained with the COPD data and their performance is evaluated when applied on a different patient population. Signals from patients undergoing bariatric surgery (BS) are used. These patients performed a controlled breathing protocol, which resulted in six different respiratory patterns. The use of these datasets for training and testing the models helps to obtain a general algorithm that is robust against changes in subjects population, and allows to generalize the classification performance for the use of normal and pathological respiration. Data augmentation (DA) and transfer learning (TL) are used to reduce the possible bias of the algorithms towards the class with the largest representation, and to optimize the models for the new, unseen data. Second, the effect of the properties of the respiration on the performance of the models is analyzed.

In this way, this study proposes a novel approach incorporating the use of DA and TL with machine learning algorithms for the quality assessment of respiratory signals from wearable devices. Also, the analysis of different breathing types using a single classification model is part of the novelty presented in this paper.

This paper is organized as follows: Section 2 describes the datasets and methodologies used in this study. In Section 3 the results are presented, and then discussed in Section 4. Concluding remarks are presented in Section 5.

## 2 Methods

### 2.1 Datasets

Two datasets were used in this study. The first one was used to pre-train the classification models, and consists of the respiratory recordings of 47 COPD patients of the Ziekenhuis Oost-Limburg (ZOL), Belgium. From the patient population, 11 were female and the mean (±standard deviation) BMI was 26.2 ± 4.9 kg/m^2^. Each patient was equipped with a wearable device to measure the BioZ, as well as a traditional wired acquisition system, which measures respiratory airflow with an airflow transducer, used as reference system. The recording of the data followed the World Medical Association’s Declaration of Helsinki on Ethical Principles for Medical Research Involving Humans Subjects. More details about this dataset can be found in (Blanco-Almazan et al., 2021; Moeyersons et al., 2021).

The second dataset consists of 72 respiratory recordings of 20 patients who underwent bariatric surgery (BS), and were in treatment at the Nederlandse Obesitas Kliniek, Netherlands. There were 16 female and the mean (±standard deviation) BMI at inclusion was 42.5 ± 3.4 kg/m^2^. The respiration of each patient was recorded using the same wearable device (BioZ) used for the COPD dataset, this time with a spirometer as the reference system. The recording of the data followed the World Medical Association’s Declaration of Helsinki on Ethical Principles for Medical Research Involving Humans Subjects.

**FIGURE 1:**
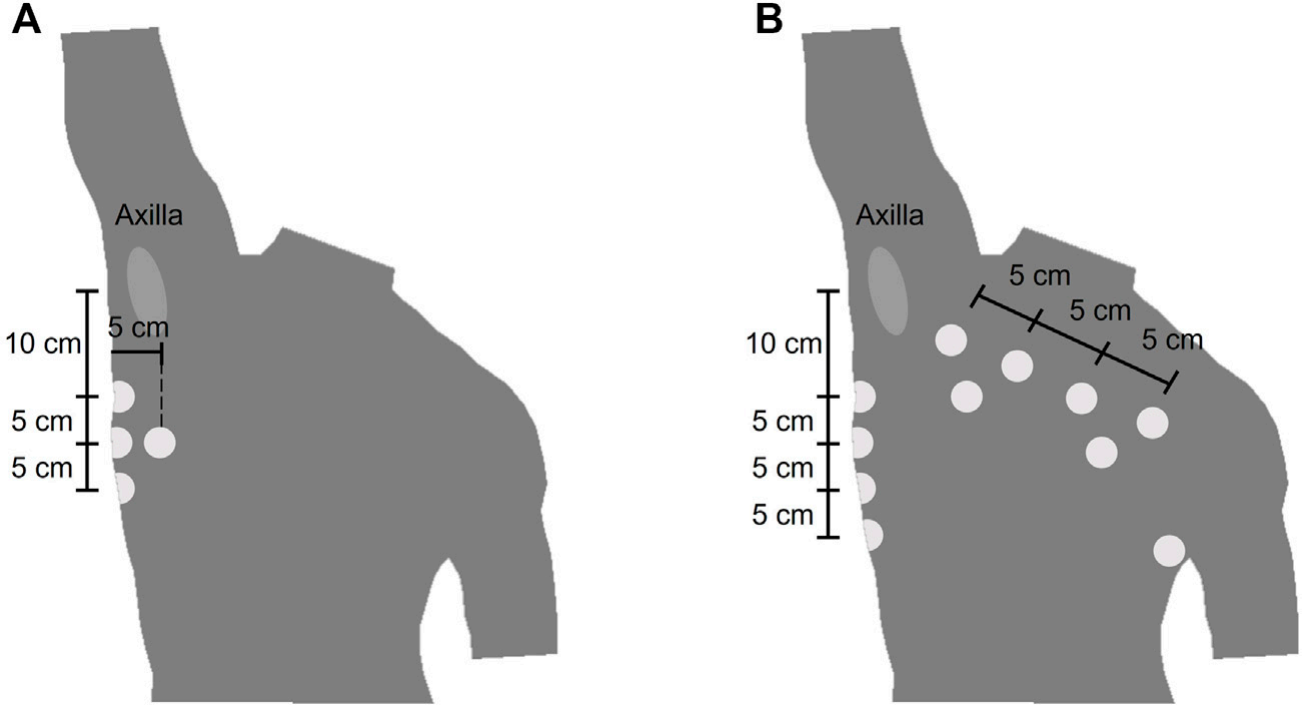
Location of the electrodes of the wearable device **(A)** Electrode placement for the COPD dataset. The electrodes were placed symmetrically from the midsternal line and only the right side is represented **(B)** Electrode placement for the bariatric surgery dataset.

The wearable device (ROBIN, Stichting imec Netherlands) recorded the BioZ signals with a sampling frequency of 16 Hz. Stress test AG/AgCl electrodes (Kendall H92SG, Covidien Inc., Walpole, MA, United States) were placed on the thorax of the subject as shown in Figure 1. Using multiplexing, four different tetra-polar configurations were created. The amplitude of the excitation current was 110* μA* at 80 kHz. The airflow for the COPD dataset was measured with a Biopac transducer (neumo-tach transducer TSD107B, Biopac Systems, Inc.), and digitalized at a sampling frequency of 10 kHz. The spirometer (TSD117A, Biopac Systems, Inc.) used a sampling frequency of 100 Hz. Further details on this equipment can be found in (Blanco-Almazan et al., 2019).

**FIGURE 2:**
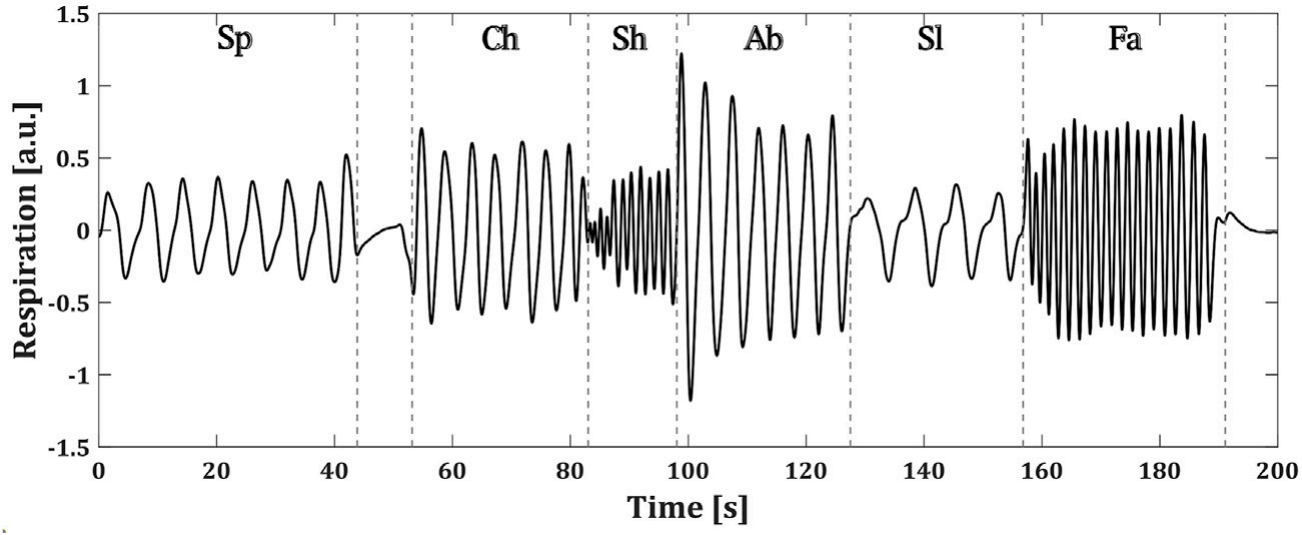
Controlled breathing protocol followed by a BS patient. Each of the breathing types are specified as: spontaneous (Sp), chest (Ch), shallow (Sh), abdominal (Ab), slow (Sl) and fast (Fa) (a.u.) stands for arbitrary units.

Each of the BS patients performed a controlled breathing protocol during the recording of their respiration. The protocol consisted of 1 minute of spontaneous breathing (Sp), followed by a period of breath holding and then five blocks of 30 seconds of chest (Ch), shallow (Sh), abdominal (Ab), slow (Sl) and fast (Fa) breathing. The pacing of the different breathing types was left to the patient’s comfort. Figure 2 shows an example of the respiratory signal of one of the patients during the followed protocol.

### 2.2 Preprocessing

Both datasets were preprocessed in the same way. First, the signals were band-pass filtered using a fourth-order Butterworth filter with cutoff frequencies at 0.05 Hz (3 breaths per minute) and 0.70 Hz (42 breaths per minute), removing the baseline changes and high frequency content not related to breathing.

The signals were then segmented as follows. For each recording, the first three and last seconds were removed due to loss of data when starting or stopping the measurements. Afterwards, the COPD recordings were divided into non-overlapping 1-min segments, resulting in 1,896 segments. In the case of the BS dataset, the breath holding period was localized and removed. After this, the recordings were divided into 30-s segments. Due to variations in the length of each respiration type, in some segments for the BS data two types of breathing were overlapped and the associated type of breathing is the one that is predominant. In total, 2,916 segments were obtained.

As one of the goals of this study was to observe the effect of different respiratory patterns on the performance of the classifiers, the segments of the BS dataset were grouped according to each of the six different breathing patterns.

### 2.3 Labeling

Supervised machine learning algorithms, such as the ones that are studied in this paper, require the ground truth of each sample for training and testing.

**FIGURE 3:**
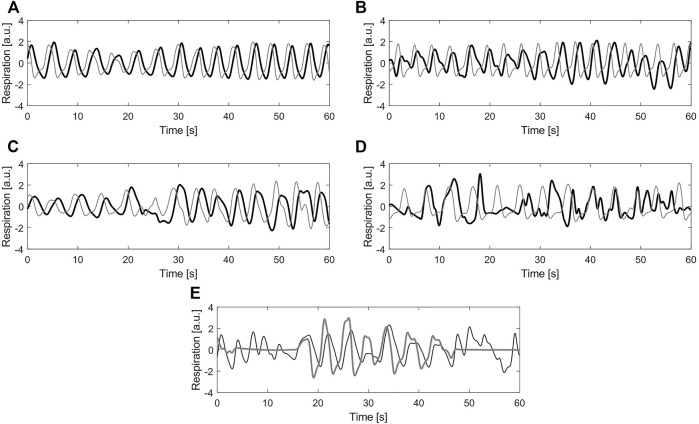
Example of different labels for quality annotation **(A)** Label 1—Excellent signal quality **(B)** Label 2—Good signal quality **(C)** Label 3—Average signal quality **(D)** Label 4—Bad signal quality **(E)** Label 5—Bad reference quality. In all plots, black line corresponds to the bio-impedance signal and the gray line to the reference signal (a.u.) stands for arbitrary units.

In this case, the ground truth of the recordings corresponds to one of the quality classes: clean or noisy. To obtain the class to which each signal belongs, four independent annotators were asked to assign a label to them. For this, the graphical user interface and the five classes defined in (Moeyersons et al., 2021) were used. The classes 1 (Excellent signal quality), 2 (Good signal quality), 3 (Average signal quality) and 4 (Bad signal quality) refer to the BioZ signal with respect to the reference system. The class 5 (Bad reference quality) is reserved for the cases where the reference signal is of bad quality due to acquisition problems, motion artefacts or signal saturation. In Figure 3 an example signal from each label is shown.

After the manual annotation of the signals, the labels for the BioZ signals were binarized, considering 1 and 2 as clean, while three and four were considered as noisy. Majority voting among annotators was performed to create a single label per signal, finding that the annotators fully agreed (4 annotators) on the 58.50% of the signals and the majority (3 annotators) agreed on the 28.09%. The Fleiss Kappa obtained for the labeling process was *κ* = 0.58, which suggested that the agreement was moderate and not at random. The segments in which no majority voting was achieved (13.41%) and the ones for which the majority voting resulted in label 5 (8.99% of the majority voting) were removed from further analysis. After this procedure, a total of 1,471 and 2,298 segments for the COPD and the BS datasets, respectively, remained.

### 2.4 Data Augmentation

It has been shown that when using machine learning approaches, the size of the train and test sets has an impact on the performance of the algorithm. The larger the training set the better the generalization of the model (Salamon and Bello, 2017; Lei et al., 2019). However, in many applications, especially those dealing with physiological data, collecting and labeling a large amount of data is not viable for various reasons, such as time limitations, ethical restrictions, or less population presenting a particular condition. One solution to this issue is to use DA (Simard et al., 2003). The principle behind DA is to generate synthetic data, using characteristics from the available data. Note that for supervised machine learning algorithms, the labels of the original data need to be preserved when generating the new data (Um et al., 2017).

In this study, four augmentation methods were applied to the BioZ signals after labeling, in order to maintain the same quality level that was originally assigned to them. The first augmentation was done by mirroring (flipping) the signal with respect to the *x*-axis and the *y*-axis. In this way, from one original signal, two new signals were obtained. This approach can be seen as an emulation of different placements of the electrodes in the skin, and does not affect the labels of the data.

The second augmentation was done by modulating the amplitude of the signals. For this, a sinusoidal modulating signal was defined with a period equal to twice the length of the original signal. In this way, the original data is modified in a way in which towards the both ends of the signal the amplitude is lower than the one at the center of the signal. In this case, when changing only the amplitude of the signal its general shape is maintained, which does not alter the labeling.

For the third method, the goal was to obtain a signal representing a slower breathing rate. To achieve this effect, the 10% of the points (5% at each end) of the original signal were removed. The time scale of the resulting segment was then assumed to be the same as the one for the original signal, meaning that if the original signal had a duration of 1 minute, the new segment was also supposed to be 1-min long. Considering this, and the fact that the new segment had less data points than the original one, it was assumed that it had a lower sampling frequency. Afterwards, it was resampled to the original sampling frequency (16 Hz), which results in a signal with a slower breathing rate. This transformation does not affect the labels of the data, given that in the guidelines for annotating the respiratory signals presented in (Moeyersons et al., 2021), the critical time frame to consider a signal of average or bad quality is the 16.6% (i.e. 10 and 5 s for the COPD and BS signals, respectively) of its length.

The goal of the final augmentation method was to obtain a signal representing a faster breathing rate. For this, two signals with the same label and from the same recording were concatenated. The resulting segment was assumed to have the same time scale than the original data but sampled at a higher frequency. Afterwards, this segment was resampled to the original sampling frequency obtaining a signal that corresponds to a faster breathing rate. The labeling of the signals is not altered, given that the concatenated signals had the same label and their general form was not altered.

**FIGURE 4:**
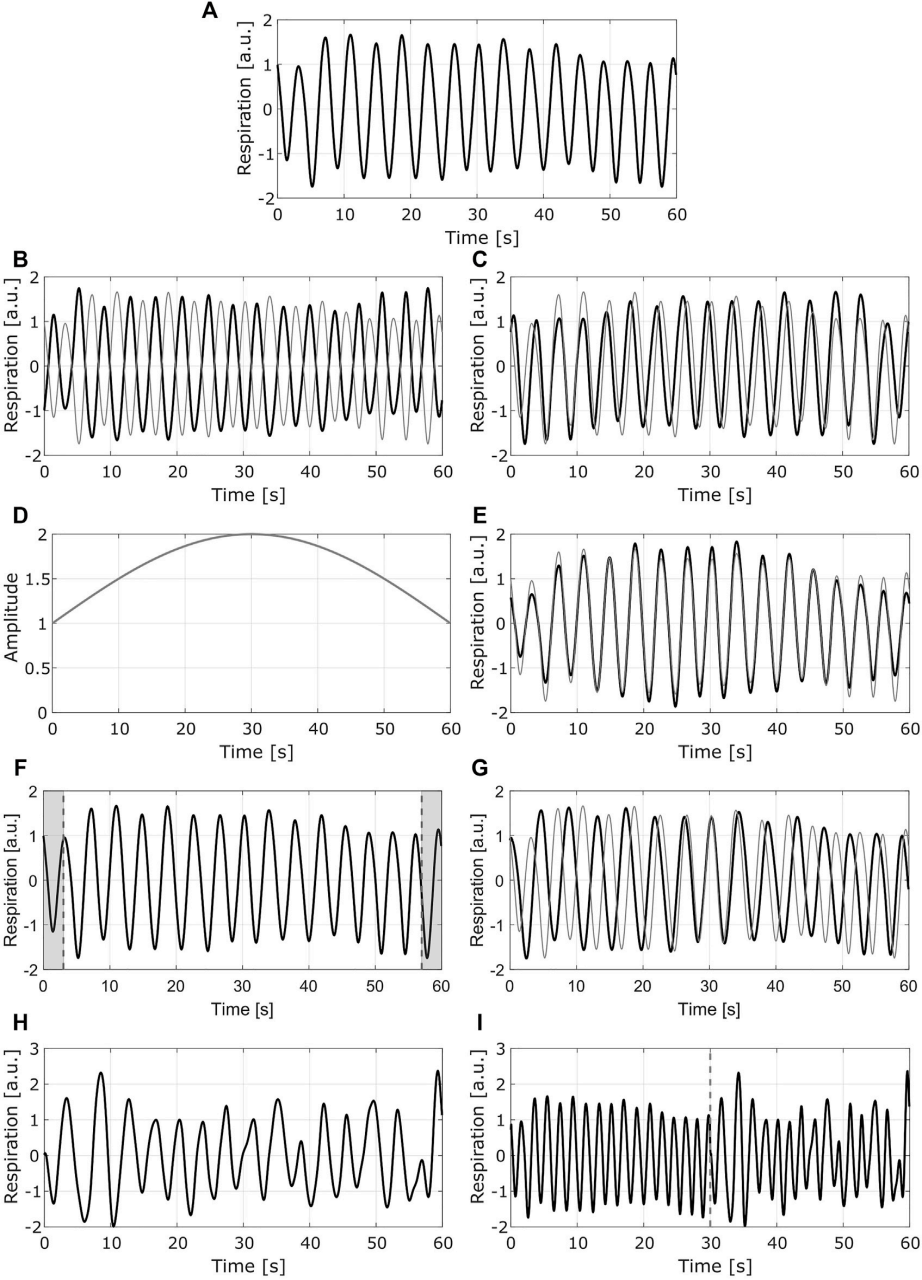
Example of the data augmentation methods. Each row presents one method **(A)** Original signal **(B)** Mirrored signal with respect to *y* axis **(C)** mirrored signal with respect to *x* axis. **(D)** Modulating signal used for the second method **(E)** normalized modulated signal (**F)** Resulting segment after removing the 10% of the points (shaded areas at each end of the signal) **(G)** resampled signal with slower breathing rate **(H)** Second original signal to be concatenated to the first one **(I)** resampled signal with faster breathing rate. In **(B) (C) (E)** and **(G)**, the gray line shows the original signal. In **(I)** the dashed gray line corresponds to the point in which the signals are concatenated; the left side corresponds to the signal in **(A)**, the right side to the signal in **(H)** (a.u.) stands for arbitrary units.

In Figure 4, an example of the methods used for DA is presented.

The previous DA methods were applied to all the signals from the quality class with less data, while they were only applied to a randomly selected number of signals of the other class.

Considering that one of the goals of this study was to analyze the effect of the characteristics of the different breathing patterns on the performance of the classifiers, for the BS data only the first two augmentations were applied. In this way, it was possible to still discriminate between the different respiratory patterns.

### 2.5 Classification

The two machine learning approaches that were evaluated in this study are described below. The input of both classifiers corresponded to the normalized signals, after subtracting the mean and dividing by the standard deviation.

#### 2.5.1 SVM

The first model was a feature-based SVM classifier. This classifier used a radial basis function kernel and its hyperparameters (i.e., box constraint and kernel scale) were selected with a Bayesian optimization technique along with five-fold cross-validation method, using the training data. The best pair of hyperparameters is obtained when the cross-validation loss is its lowest, and these are then used to train the optimal SVM model.

The features used with this model were computed from the whole segment and for 15-s sub-segments. In the case of the COPD data, the segments had a length of 60 s, while for the BS data the length of the signals was 30 s. The sub-segments of the COPD data were obtained without overlap. In contrast, the sub-segments of the BS data were obtained with a 10-s overlap.

The features were calculated from the auto-correlation function (ACF): amplitude of the first peak (Ap1), amplitude of the second peak (Ap2) and ratio between these two peaks (Ap1/Ap2); and from the power spectral density (PSD): bandwidth, frequency of the lower (*f*_*low*_) and upper (*f*_*up*_) bounds of the bandwidth, and the normalized power in this band. The five more informative features, common to all the signals, were selected as in (Moeyersons et al., 2021), using a maximum relevance minimum redundancy (MRMR) algorithm. From the ACF, the most relevant features were the Ap1 of the whole segment and the standard deviation of the Ap1 of the sub-segments. From the PSD, the most relevant features were: the *f*_*low*_ of the whole segments, the mean bandwidth of the sub-segments and the mean normalized power of the sub-segments.

More information on this model can be found in (Moeyersons et al., 2021).

#### 2.5.2 CNN

The second classifier was a 1-dimensional CNN. The architecture of the network consisted of two blocks, each with two convolutional layers, followed by a global average pooling and ending with a fully-connected output layer with a softmax activation function.

Each of the two convolutional layers of the first block had 10 filters with a kernel size of 32 samples. The stride of these filters was of two samples and the padding in the borders was defined as the same end values. These layers had a ReLU activation function.

The output of the first block was then passed to the second block. The two convolutional layers of this block had the same hyperparameters of the ones from the previous block, but with only five filters instead of 10. This was done to ensure a resulting feature map with five features, analogous to the SVM approach.

This feature map was received by the average global pooling layer, which was used to generate a single feature vector by averaging the map over the temporal axis. Along with the characteristic behaviour of the convolutional layers, the robustness of this layer allowed to use the network with input signals of different sizes.

Finally, the feature vector was fed to the fully-connected layer, obtaining as output the classification probabilities for the two classes.

For more information regarding the architecture of this model, please refer to (Moeyersons et al., 2021).

### 2.6 Transfer Learning

One of the main assumptions of machine learning algorithms used for classification problems is that both the training and testing data have the same distribution and the same feature space. These assumptions, however, do not always hold in real life applications. An alternative to tackle this issue is TL (Weiss et al., 2016). With TL, a new classification problem is solved by using as a starting point an existing solution from a similar problem. Thus, the new classification problem requires less training data to obtain a robust solution.

In this study, the TL approach described in (De Cooman et al., 2020) was used for the SVM classifier. In this approach, an adapted model is generated by modifying the objective function of the SVM. For this, the classification error of the new data (BS signals) is minimized, as well as the dissimilarity between the original and the adapted models. One assumption of this approach is that the same features used for training the original model with the original data also describe the new data in which the adapted model is going to be applied. Considering this, the same features that were found to be the most relevant for the COPD data were computed and used with the BS data. Moreover, TL was applied to the original model in order to optimize the SVM for each breathing type.

For the CNN, the same principle presented in (Nanni et al., 2020) was used. In this approach, the adapted model is generated by copying the architecture and the weights of the pre-trained CNN. In a first step, the weights of all layers apart from the actual classification layer are fixed and then the classification layer is retrained. After, a fine-tuning (FT) step is added. In this step, the previously fixed weights are un-fixed and all the weights of the adapted model are retrained. The retraining is done setting a low learning rate and a small number of epochs to prevent modifying significantly the weights of the model. The only assumption of this approach is that the signals from the training and testing datasets should have the same sampling frequency. TL including FT was applied to the original CNN to obtain an adapted model for each breathing type.

### 2.7 Performance Evaluation

The models were tested with a cross-validation approach. The division was done by taking all the segments of the 70% of the recordings of the BS data for the training step of TL. The remaining 30% was used for testing the models. In order to assess the generalization capability of the models, this division was done 10 times at random. The same splits were used for both models with and without TL.

The area under the ROC curve (AUC) was used as the metric to evaluate and compare the performance of the models.

Significant differences between the models were evaluated with a Wilcoxon signed rank test (significant if *p* < 0.05) to assess the utility of TL and DA for the BS dataset.

### 2.8 Training and Testing

The classifiers were pre-trained with two strategies. The first one was to train the classifiers with all the available segments from the COPD data without DA. The second one was to train the classifiers with all the segments from the COPD data including DA.

The models obtained with these two strategies were tested on the BS data. Then, TL was applied to the models pre-trained with the COPD data with DA, using the BS with DA. This was done to ensure that when re-training the models during TL, the new data was sufficient for the task.

In this way, for each breathing type, three results were obtained, further referenced as Original (model pre-trained with COPD data without DA), DA (model pre-trained with COPD data including DA) and TL + DA (model pre-trained with COPD data including DA and TL).

## 3 Results

The total number of segments after labeling and removing the ones with bad reference quality, for each dataset, is presented on the left side of Table 1. As can be observed, for the COPD there were more clean segments available, while for all the breathing types of the BS dataset there were more noisy segments. On the right side of the table the total number of segments for each data group after applying DA is presented.

**TABLE 1:** Overview of the datasets, indicating the number of segments in each class. The suffix corresponds to the type of breathing imposed during the respiratory protocol. On the left, the total number of segments before data augmentation (DA). On the right, the segments after DA.

Group	Before DA			After DA		
	Clean	Noisy	Total	Clean	Noisy	Total
COPD	1,118	353	1,471	2072	2081	4,153
BS-Sp	202	240	442	808	807	1,615
BS-Ch	181	317	498	724	722	1,446
BS-Sh	181	305	486	724	722	1,446
BS-Ab	208	256	464	832	832	1,664
BS-Sl	83	216	299	332	330	662
BS-Fa	26	83	109	104	104	208

**FIGURE 5:**
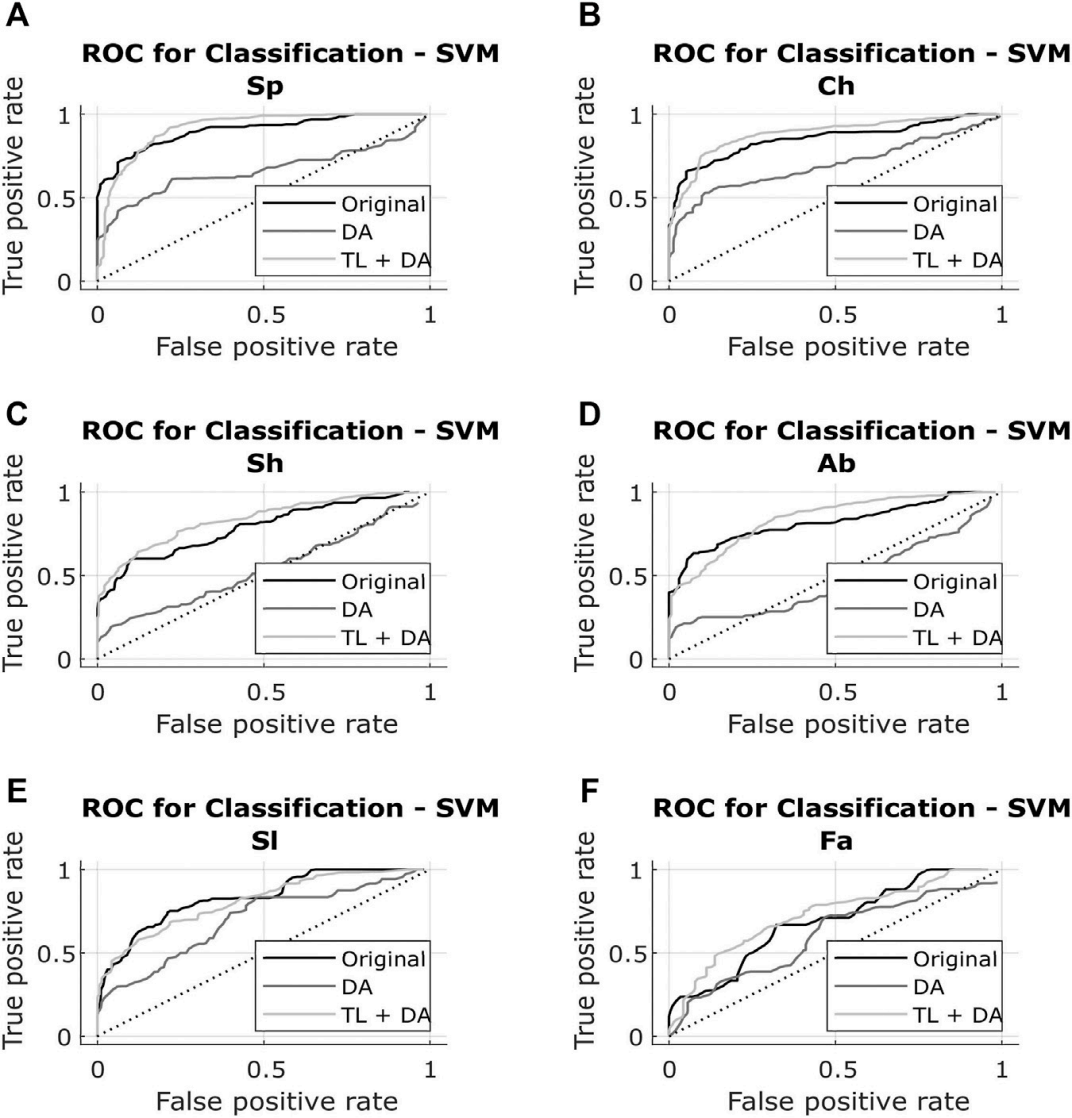
ROC of the SVM models for each of the sub-groups of the BS dataset **(A)** Spontaneous breathing (Sp) **(B)** Chest breathing (Ch) **(C)** Shallow breathing (Sh) (**D)** Abdominal breathing (Ab) **(E)** Slow breathing (Sl) (**F)** Fast breathing (Fa). The curves present the median ROC for each model, in black the original, in dark gray the SVM-DA and in light gray the SVM-TL + DA. The dotted line corresponds to the random guess. It is observed that in all the breathing types the DA model presents a worse behavior than the other models, and that the TL + DA model shows a similar behavior to the Original one.

**FIGURE 6:**
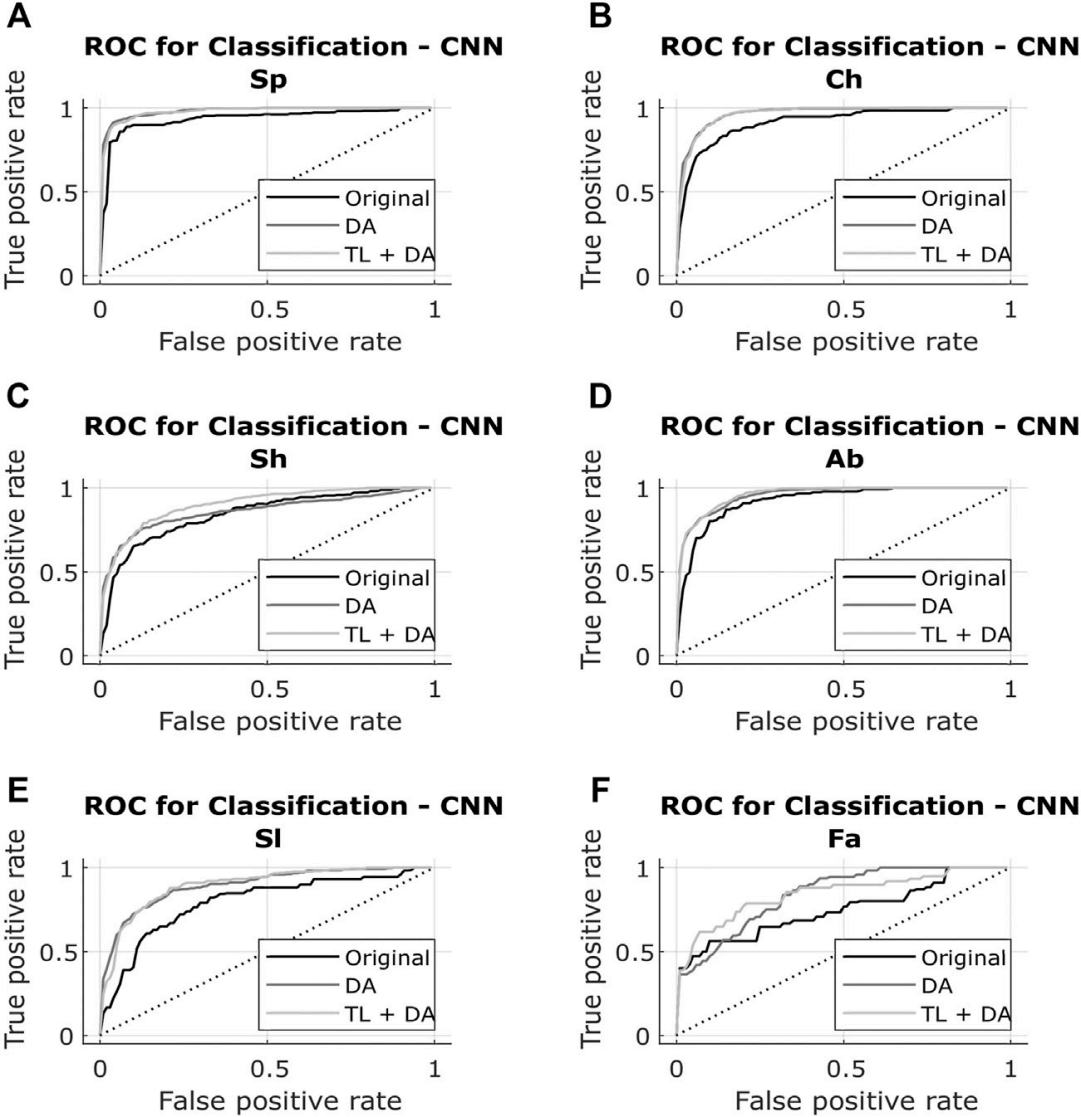
ROC of the CNN models for each of the sub-groups of the BS dataset **(A)** Spontaneous breathing (Sp) **(B)** Chest breathing (Ch) **(C)** Shallow breathing (Sh) **(D)** Abdominal breathing (Ab) **(E)** Slow breathing (Sl) **(F)** Fast breathing (Fa). The curves present the median ROC for each model, in black the original, in dark gray the CNN-DA and in light gray the CNN-TL + DA. The dotted line corresponds to the random guess. It is observed that the models DA and TL + DA exhibit a better behavior than the Original one, while behaving similar to each other.

The results obtained with both classification approaches are presented in Table 2. Results are indicated as median AUC [25th percentile - 75th percentile]. The values that are in bold correspond to the best results obtained with each classifier for each breathing type. The best results were defined as the ones with the higher AUC, which presented a significant (*p* < 0.05) improvement compared to the original results. In case of non-significant differences, the results with the smaller interquartile range were preferred. In Figures 5, 6, the median of the ROC curves are presented for each of the classifiers, for the different breathing types. The original model is presented in black, the DA model in dark gray, and the TL + DA model in light gray. For the SVM, in all the breathing types it was observed that the DA model presented a worse behavior than the other models, and that the TL + DA model showed a similar behavior to the Original one. In contrast, for the CNN, the models DA and TL + DA showed a better behavior than the Original one, but they were similar to each other.

**TABLE 2:** Performance of the machine learning models for each of the sub-groups of the BS dataset. The results are presented as median AUC (25th percentile—75th percentile) (%). On the left side, the results for the SVM. On the right side, the results for the CNN. The best results (i.e., higher AUC) for each model for each breathing type are in bold.

—	SVM			CNN		
	Original	DA	TL + DA	Original	DA	TL + DA
Sp	91.03	66.92	**93.58^++^**	93.43	98.32***	97.92**^,^*
	[88.29–93.10]	[63.35–70.41]	[91.03–94.85]	[92.49–93.81]	[96.69–98.81]	[97.24–98.21]
Ch	84.80	70.84	**89.32^++^**	90.56	96.12***	96.10***
	[83.24–91.88]	[67.31–73.18]	[86.51–91.40]	[89.54–93.08]	[95.94–96.70]	[95.68–96.29]
Sh	79.68	52.18	**84.75^++^**	83.86	84.84	90.22**^,+^
	[73.89–83.04]	[49.47–59.64]	[79.34–88.06]	[80.06–89.73]	[82.88–89.45]	[87.89–92.82]
Ab	81.70	47.90	**84.80^++^**	91.74	94.74**	95.13*
	[77.78–86.18]	[43.55–51.21]	[83.45–87.77]	[91.07–93.92]	[93.01–96.23]	[93.5–97.25]
Sl	85.65	71.73	**81.29^++^**	78.12	90.54**	89.26**
	[79.41–86.46]	[66.52–74.34]	[79.20–83.92]	[72.89–86.83]	[87.77–93.51]	[87.23–93.05]
Fa	69.09	61.25	**74.05^+^**	73.02	84.65*	83.41*
	[59.64–75.69]	[45.86–70.56]	[65.71–76.64]	[71.82–77.78]	[83.00–87.50]	[74.74–92.85]

Breathing patterns: Sp, spontaneous; Ch, chest; Sh, shallow; Ab, abdominal; Sl, slow; Fa, fast.

Significant results compared to the Original model, *p < 0.05, **p < 0.01, ***p ≪ 0.01.

Significant results compared to the DA, model, ^+^ *p* < 0.05, ^++^ *p* ≪ 0.01.

In the case of the SVM, in order to compare the results obtained with all the models, it was assumed that the best five features selected for the pre-training of the Original model were still the best for the new models. However, it can be seen that the performance of the DA model decreased in comparison to the Original one. Nevertheless, it can be noted that TL + DA improved significantly (*p* < 0.05) the performance compared to the DA model for all the breathing types.

In contrast, in the case of the CNN the performance of the DA and TL + DA models improved significantly (*p* < 0.05) with respect to the Original model, with the only exception of Sh breathing. However, TL showed a significant improvement of the performance compared with the DA model only for the Sh breathing.

A comparison with the heuristic model presented by Charlton et al. (2021) was also performed to evaluate the advantages that using machine learning methods supposes when assessing the quality of respiratory signals. Charlton’s signal quality index classify segments into high or low quality based on the variation on breath duration, the definition of peaks and troughs, and the similarity of the morphology of the breaths. The comparison of the accuracy, sensitivity and specificity of the classification of each breathing type are presented in Tables 3–5, respectively. Results are indicated as median [25th percentile—75th percentile]. The values that are in bold correspond to the best results obtained with each classifier for each breathing type, that were also higher than the heuristic model.

**TABLE 3:** Performance of the machine learning models for each of the sub-groups of the BS dataset. The results are presented as median accuracy (25th percentile—75th percentile) (%). On the left side, the results for the SVM. On the middle, the results for the CNN. On the right side, the results using the heuristic method in Charlton et al. (2021). The best results of each model that performed better than the heuristic approach for each breathing type are in bold.

—	SVM			CNN			Heuristic
	Original	DA	TL + DA	Original	DA	TL + DA	
Sp	83.13	65.63	**87.23**	87.41*	81.21	92.41*	84.03
	[81.82–84.91]	[58.57–70.59]	[85.64–89.53]	[86.33–88.1]	[79.43–84.35]	[91.61–93.20]	[82.35–86.32]
Ch	82.10	66.25	**83.55**	85.72*	73.20	89.06*	80.97
	[80.60–84.55]	[64.08–68.37]	[81.87–85.20]	[82.43–87.41]	[71.59–74.88]	[88.32–89.68]	[79.05–83.58]
Sh	**80.18**	70.31	77.71	74.66	65.01	**82.60**	79.26
	[69.88–84.21]	[59.04–77.68]	[71.78–83.26]	[71.33–79.47]	[61.00–66.43]	[80.04–85.00]	[71.08–87.50]
Ab	72.21	61.13	75.57	85.61*	70.62	87.33*	76.51
	[69.49–76.79]	[57.63–66.27]	[72.00–76.76]	[83.46–87.23]	[69.12–73.01]	[85.66–89.62]	[68.18–79.52]
Sl	80.19*	72.76	69.93	76.79	63.02	82.96*	73.82
	[77.33–85.14]	[67.39–82.43]	[67.26–72.48]	[73.03–80.21]	[60.98–66.50]	[79.07–86.19]	[72.55–78.57]
Fa	**72.67**	69.62	72.35	**80.33**	52.42	75.39	69.62
	[61.90–87.50]	[61.90–83.33]	[68.35–76.92]	[71.43–89.29]	[42.11–61.70]	[63.89–83.64]	[61.90–83.33]

Breathing patterns: Sp, spontaneous; Ch, chest; Sh, shallow; Ab, abdominal; Sl, slow; Fa, fast.

Significant results compared to the Heuristic method, *p < 0.05.

**TABLE 4:** Performance of the machine learning models for each of the sub-groups of the BS dataset. The results are presented as median sensitivity (25th percentile—75th percentile) (%). On the left side, the results for the SVM. On the middle, the results for the CNN. On the right side, the results using the heuristic method in Charlton et al. (2021). The best results of each model that performed better than the heuristic approach for each breathing type are in bold.

—	SVM			CNN			Heuristic
	Original	DA	TL + DA	Original	DA	TL + DA	
Sp	63.92	13.42	83.48	76.32	61.26	**91.69** **^∗^**	85.90
	[61.11–68.75]	[8.57–19.44]	[80.43–89.42]	[71.43–76.81]	[57.65–67.86]	[89.13–93.37]	[81.58–91.30]
Ch	55.72	9.29	**85.87** **^∗^**	68.07	48.12	**89.86** **^∗^**	64.17
	[46.67–59.52]	[6.82–11.11]	[80.00–88.89]	[63.64–74.14]	[40.00–49.60]	[87.22–90.69]	[57.78–70.37]
Sh	35.61	2.17	**81.41** ^∗^	46.49	34.45	**81.57** ^∗^	52.78
	[33.33–47.22]	[0.00–5.56]	[74.22–87.14]	[41.94–48.48]	[31.05–35.00]	[74.48–86.57]	[45.71–66.67]
Ab	42.64	10.91	**78.84** ^∗^	77.62*	46.50	**86.46** ^∗^	62.50
	[40.91–43.75]	[0.00–12.50]	[71.88–81.58]	[74.32–82.09]	[44.64–49.32]	[84.33–90.82]	[55.56–64.00]
Sl	34.31	8.69	**64.99** ^∗^	35.57	24.55	**79.56** ^∗^	39.09
	[28.57–42.86]	[0.00–17.65]	[58.33–68.75]	[33.33–42.11]	[18.42–28.41]	[76.14–83.62]	[33.33–41.18]
Fa	21.11*	5.00	**77.68** ^∗^	35.42	10.36	**61.88** ^∗^	10.56
	[14.29–33.33]	[0.00–13.33]	[75.00–100.00]	[28.57–40.00]	[10.00–12.50]	[47.50–84.38]	[0.00–14.29]

Breathing patterns: Sp, spontaneous; Ch, chest; Sh, shallow; Ab, abdominal; Sl, slow; Fa, fast.

Significant results compared to the Heuristic method, *p < 0.05.

**TABLE 5:** Performance of the machine learning models for each of the sub-groups of the BS dataset. The results are presented as median specificity (25th percentile—75th percentile) (%). On the left side, the results for the SVM. On the middle, the results for the CNN. On the right side, the results using the heuristic method in Charlton et al. (2021). The best results of each model that performed better than the heuristic approach for each breathing type are in bold.

—	SVM			CNN			Heuristic
	Original	DA	TL + DA	Original	DA	TL + DA	
Sp	**95.81** ^∗^	100.00	88.37*	97.10*	**99.57** ^∗^	93.72*	84.98
	[91.89–98.33]	[100.00–100.00]	[84.62–94.62]	[96.30–97.59]	[99.29–99.61]	[92.53–95.24]	[78.13–85.42]
Ch	**97.40** ^∗^	100.00	81.60	94.44*	**98.86** ^∗^	90.35	90.98
	[96.88–98.39]	[100.00–100.00]	[79.75–84.38]	[93.91–96]	[98.47–100.00]	[87.94–93.51]	[88.71–92.19]
Sh	**99.43** ^∗^	100.00	70.54	96.74*	**99.08** ^∗^	87.33	89.53
	[95.00–100.00]	[98.73–100]	[67.57–74.32]	[95.71–97.41]	[98.9–99.62]	[85.61–88.5]	[88.68–92.31]
Ab	**98.44** ^∗^	100.00	72.53	91.15*	**98.87** ^∗^	87.62*	83.68
	[96.30–100.00]	[100.00–100.00]	[70.10–77.65]	[90.16–94.20]	[97.17–99.61]	[86.57–90.71]	[81.25–88.89]
Sl	**98.01** ^∗^	100.00	74.26	91.48	100.00	87.17	89.29
	[97.14–100.00]	[100.00–100.00]	[68.82–81.82]	[89.58–93.90]	[100.00–100.00]	[83.53–89.36]	[88.24–95.24]
Fa	100.00	100.00	55.84	100.00	100.00	85.52	100.00
	[100.00–100.00]	[100.00–100.00]	[50.00–73.91]	[96.30–100.00]	[100.00–100.00]	[78.26–92.59]	[100.00–100.00]

Breathing patterns: Sp, spontaneous; Ch, chest; Sh, shallow; Ab, abdominal; Sl, slow; Fa, fast.

Significant results compared to the Heuristic method, *p < 0.05.

## 4 Discussion

In this study, the performance of two pre-trained machine learning classifiers for the quality assessment of respiratory signals was evaluated on a dataset obtained from patients performing a respiratory protocol in which different breathing rates were imposed. To improve the performance of the classifiers, two techniques were used, namely Transfer Learning (TL) and Data Augmentation (DA).

It was noted that for the Slow (Sl) and Fast (Fa) breathing rates there were not as many data as for the other breathing types. In a first instance, there were less Fa data due to variations in the length of the raw data, which affected the segmentation of the last part of the signals, making it more difficult to obtain 30-s segments of the last breathing type. Second, for these two types of breathing the annotators found it more difficult to reach an agreement in the labeling (*κ* = 0.49 for Sl, and *κ* = 0.36 for Fa). Besides, the quality of the reference signals was not good enough in the 18.97 and 32.30% of the data for which the majority voting was obtained for the Sl and Fa, respectively. The low quality of the reference signal was due to disconnection of the spirometer and breath holding periods during the protocol. This might represent a challenge to future wearable monitoring developments, in particular if they are meant to be used in space and in early prediction of health deterioration, as they should ensure the good quality of signals with a wide range of breathing rates.

It was also observed that the distributions of noisy and clean segments in the BS and COPD datasets were different. While the BS dataset contained more noisy than clean signals, the opposite was observed in the COPD dataset. This behavior could be related with three factors. First, the higher BMI in the BS dataset has an impact in the impedance of the thorax making the signals more noisy. Second, the respiratory protocols followed by the patients could have an impact in this distribution. Third, as was shown in Figure 1, the electrode placement was different for each group, which could also affect the measurement of the impedance. However, it is suggested to investigate further the cause of these distributions differences as it can help to design a more robust device for vital signs monitoring.

When pre-training the models with the COPD data without using DA, it was found that for the Sh and Fa breathing types, the performance of the SVM was worse than for the other breathing types (median AUC <80%). In the case of the CNN, the performance was worse (median AUC <80%) for the Sl and Fa types. The reasons behind this behavior could be the different distribution of the classes in the training and testing sets, and the combination of morphological characteristics with the different breathing rates of the signals in the testing set in comparison with the training data. This is more evident when comparing the results obtained in the present study with the ones presented in (Moeyersons et al., 2021). In the latter, the SVM and CNN were trained and tested in the same COPD dataset. The average AUC obtained in that case is comparable to the results obtained for Sp, Ch and Ab data, which present a more similar breathing rate to the COPD data (normal breathing rate between 8 and 14 breaths per minute).

To overcome these challenges, it was decided to pre-train the models with the COPD data after using DA. The models then were tested in the original BS dataset. With this, the amount of clean and noisy signals in the COPD data was balanced, and different characteristics were included (different breathing rates and changes in amplitude). In this case, it was found that for the SVM, the performance of all breathing types decreased in comparison with the Original model. As mentioned before, the five more relevant features obtained from the original dataset were used as well for this analysis. However, the inclusion of the synthetic data to the training set could have altered the relevance of all the features and the best ones could have changed. The effect of changing the selection of the best five features was not explored in this study, and it is proposed as future research. The selection of the best features for the SVM classifier is a critical design variable in the development of decision support systems, as it can impact the performance of the model. It is also worth mentioning that in this study only five features were selected, however, as was shown in (Moeyersons et al., 2021), selecting more or less features also have an impact on the performance of the SVM. This effect should be also investigated further when working with TL, DA and more importantly with different types of signals for vital signs monitoring applications in extreme and remote environments.

In contrast, the performance of the CNN for the pre-training with the COPD data after DA improved significantly. It is known that the performance and the generalization capabilities of machine learning models are highly dependent on the quantity of data available for training (Salamon and Bello, 2017; Lei et al., 2019; Iwana and Uchida, 2021). In the case of the CNN, it performs an automatic feature selection based on the input signals, which implies that if a larger and more diverse set is available, the selection of the relevant features will be improved and the performance will be better. The notable difference between the SVM-DA and the CNN-DA is explained by the way in which the features for the classification are selected, being pre-defined for the SVM and automatically for the CNN.

As a final step to help improve the performance of the classifiers, TL was applied to the pre-trained models with the COPD data with DA. To ensure that the re-training set for TL was balanced, the BS data with DA were used. In this case, it was found that the performance after applying TL was consistently better than for the DA model for all the respiration types for SVM. However, it did not improve significantly with respect to the Original model. This could also be explained by the fact that when including synthetic data in the BS groups, the feature space changes and alters the selection of the best features. For CNN, the performance improved significantly compared with the Original model, but not compared with the DA model. One explanation for this could be that after including the DA in the training set, the inclusion of the DA in the BS data does not provide new and better information for the classification.

The effect of these two approaches is more noticeable when compared to the results presented by Rozo et al. (2021), where TL was applied to the models pre-trained with the COPD data without DA. In that case, SVM presented a higher improvement than CNN. The results obtained in the present study show that the inclusion of DA before applying TL reduces the improvement of performance in the SVM. In contrast, the inclusion of DA with and without applying TL have a bigger effect in the performance of the CNN, than only using TL.

In general, it could be seen that DA is a good alternative to improve the performance of machine learning models in which the features selection is done automatically. In contrast, when the features are computed a-priori and the best ones to be used are hand-picked, the best alternative could be the use of TL. Nevertheless, it is still important to research more in depth the dependencies of the performance of the classifier on DA and TL, as in this study only a limited patient population was used.

Despite the general improvement of both classifiers after using DA and TL, it could be seen that the specific performance for Sh, Ab, Sl and Fa breathing types is lower compared to Sp and Ch. This could be due to the fact that the changes of morphology and breathing rates included in the pre-training set after DA, were not enough to characterize the new signals in which the models were tested. More research is needed to characterize correctly the combinations of morphology and variation of breathing rates that consistently challenge the performance of the classifiers. In this way, it will be possible to have a better performance with a wider range of respiratory signals obtained with wearable devices for different applications. However, it is important for the model to achieve a balance between generalization capabilities and good performance, to fulfill the goal of using it with any new data. For this, it is proposed to collect and study a more diverse cohort of data in terms of length of the segments and protocols followed by the patients, to ensure that the models presented in this manuscript for the quality assessment of respiratory signals are sufficiently robust and general.

In addition, the performance of the machine learning models was compared with the performance of the heuristic method for signal quality index proposed by Charlton et al. (2021). It was found that, in general, the machine learning models presented a better performance, and it was improved even further when using DA and TL. This findings are in line with the comparison presented by Moeyersons et al. (2021), where it was found that for the COPD data quality assessment, the machine learning models performed significantly better than the heuristic model. This could serve as a basis for the selection of machine learning models over heuristic models when assessing the quality of respiratory signals obtained from wearable devices.

## 5 Conclusion

In this work, the quality assessment of respiratory signals obtained from wearable sensors was studied. The results presented in this study showed that with pre-trained machine learning classifiers in conjunction with data augmentation and transfer learning, it is possible to properly identify clean and noisy respiratory BioZ signals. CNN performed overall better than SVM when using DA, but the effect of TL was more noticeable in SVM.

For both classifiers, however, the results showed a lower performance for the breathing types whose morphology and imposed breathing rates differed the most from the spontaneous breathing of the patients in which the models were pre-trained.

These findings could be beneficial for the steps of data processing and connection with decision support systems when designing new bio-monitoring devices for space exploration.

## Data Availability

The data that support the findings of this study are available on reasonable request to the corresponding author. The data are not publicly available due to them containing information that could compromise research subject privacy.
